# Effects of Sodium Hypochlorite Washing on Eggshell Membrane Integrity, Microbial Decontamination, and Trace Element-Related Dietary Risk in Poultry and Duck Eggs

**DOI:** 10.1007/s12011-026-05274-4

**Published:** 2026-08-10

**Authors:** Mahfuzur Rahman, Claudia Ionita, Mohammad Amirul Islam, Kay Cooksey, Omer Sadak

**Affiliations:** 1 Department of Food, Nutrition, and Packaging Sciences, Clemson University, Clemson, SC 29634 USA; 2 Institute of Nuclear Science & Technology, Atomic Energy Research Establishment, Bangladesh Atomic Energy Commission, Ganakbari, Ashulia, Dhaka 1349 Bangladesh

**Keywords:** Food safety, Elemental profiling, ATR–FTIR spectroscopy, SEM–EDS, Exposure assessment

## Abstract

This study examined baseline trace element exposure in poultry and duck eggs consumed in Bangladesh and assessed the impact of sodium hypochlorite (NaOCl) washing on microbial decontamination and eggshell membrane integrity. Fifteen elements were measured in yolk, albumen, and whole egg samples using instrumental neutron activation analysis (INAA) and atomic absorption spectrometry (AAS). Most elements were below maximum allowable concentrations; however, Pb and Cr exceeded permissible limits in some whole egg samples. Despite this, exposure-related risks, including estimated daily intake (EDI), target hazard quotient (THQ), hazard index (HI), and target carcinogenic risk (TCR), remained within acceptable limits for both adults and children. Multivariate analyses, such as principal component analysis (PCA) and hierarchical cluster analysis (HCA), revealed distinct clustering patterns and relationships among elements across different egg parts and species. In addition, eggs were treated with 0–200 ppm NaOCl to evaluate microbial reduction and structural effects on the eggshell membrane. NaOCl treatment significantly reduced total bacterial and fungal loads, with 150 and 200 ppm reducing cultivable microorganisms to below the detection limit (10 CFU g⁻^1^). Moderate NaOCl levels effectively enhanced microbial safety, while higher concentrations caused oxidative modifications and structural deterioration of the eggshell membrane.

**Supplementary Information:**

The online version contains supplementary material available at https://doi.org/10.1007/s12011-026-05274-4.

## Introduction

Elemental toxicology has become a growing concern for both the environment and the food chain. These toxic elements persist in soils and water, resist degradation, and accumulate in animal tissues through the food chain, posing risks to animals and humans even at low concentrations [4]. Anthropogenic activities such as mining, urbanization, industrialization, fossil fuel combustion, and improper waste disposal significantly contribute to environmental contamination [24, 40]. Essential elements, such as iron (Fe), copper (Cu), and zinc (Zn), are required for oxygen transport, metabolism, and energy production [46]. In contrast, non-essential elements, including lead (Pb), chromium (Cr), and cadmium (Cd), have no physiological role and may be toxic even at trace levels in the body [52]. Long-term exposure to these elements may cause neurological damage, skeletal disorders, reproductive problems, and liver injury [51].

The poultry industry plays an important role in the rural economy of developing countries such as Bangladesh due to its low capital requirements. It supports nearly six million livelihoods, with many women involved in poultry farming [4]. Poultry and duck eggs are also essential for reducing micronutrient deficiencies and improving maternal and child health [38]. According to FAO and BBS [1], the average Bangladeshi consumes 0.3–0.4 eggs per day, which corresponds to approximately 110–146 eggs per person per year, compared with approximately 290 eggs per person annually in the United States and 180–230 eggs per person annually in many Asian countries [20]. Eggs may become contaminated by microorganisms, chemical residues, or physical hazards, leading to quality deterioration and food safety risks [28]. Eggs possess a porous calcium carbonate shell that allows the transfer of gases, moisture, microorganisms, and environmental pollutants [43]. The protein-rich inner membranes are readily oxidized and physically damaged during handling and storage [8]. Eggs also serve as important functional ingredients in the food industry due to their foaming and emulsifying properties [42]. Nevertheless, eggs are frequently contaminated by pathogens such as *Salmonella enterica*, *Escherichia coli*, and *Staphylococcus aureus* [5, 37], which may originate from infected hens or contaminated environments, posing significant food safety and economic challenges [24].

After collection from farms, eggs must be properly cleaned; however, there is no universal standard for egg-cleaning practices. In many rural farming systems, eggs are often washed only with water due to limited access to testing facilities [43]. Washing eggs can reduce microbial contamination and improve their appearance [10]. Eggs can be cleaned using various approaches, including chemical, physical, and advanced technologies [2]. Chemical sanitizers such as sodium hypochlorite, chlorine dioxide, hydrogen peroxide, and organic acids reduce microbial contamination through oxidative or acidic mechanisms [6, 32]. Advanced technologies, such as ozone treatment and UV-C irradiation, can inactivate microorganisms without leaving chemical residues, while physical methods, such as dry brushing, mechanically remove dirt [2, 61]. Sodium hypochlorite (NaOCl) is widely used for egg sanitation because it is inexpensive and effective. Free chlorine released from NaOCl damages microbial cell walls, proteins, and metabolic pathways [18]. However, excessive use of NaOCl may compromise egg safety. In aqueous solution, sodium hypochlorite forms hypochlorous acid, a strong oxidizing species that can damage microbial membranes, proteins, enzymes, and nucleic acids. Its antimicrobial efficacy is influenced by available chlorine concentration, pH, temperature, contact time, organic load, and the initial microbial population [29, 42]. Although appropriately controlled NaOCl washing can reduce eggshell contamination, excessive concentration or exposure may modify the cuticle and shell surface, increase pore exposure, and influence moisture loss and internal egg quality during storage. Previous egg studies have therefore emphasized the need to balance microbial inactivation with preservation of shell microstructure and quality attributes [39, 61]. According to the USDA, 100–200 ppm NaOCl is permitted for egg decontamination [11, 28]. In contrast, the European Union and the United Kingdom prohibit NaOCl washing for the production of Grade A eggs under EC regulations [57]. Japan allows 150 ppm NaOCl for industrial egg processing [18].

Previous studies have generally evaluated egg elemental contamination, microbial decontamination, storage quality, or shell structure as separate research questions. Limited information is available from an integrated framework combining baseline multi-element profiling and dietary-risk assessment with NaOCl-mediated microbial reduction, physicochemical quality, and eggshell membrane chemistry and morphology. In addition, direct comparisons between poultry and duck eggs remain limited. Therefore, this study simultaneously evaluated 15 elements and associated dietary-risk indices while examining the microbial, physicochemical, ATR–FTIR, SEM, and EDS responses of poultry and duck eggs to 0–200 ppm NaOCl. This integrated approach provides new insight into the balance between decontamination efficacy, membrane integrity, egg quality, and exposure-related safety.

## Materials and Methods

### Chemicals and Reagents

All chemicals were analytical or trace-metal grade. Sodium hypochlorite (NaOCl, 12–15% available chlorine) and nitric acid (HNO₃, 65%, TraceMetal™ grade) were obtained from Merck (Darmstadt, Germany). Hydrogen peroxide (H₂O₂, 30%) and potassium bromide (KBr, FTIR grade) were obtained from Sigma-Aldrich (USA). Certified reference materials SRM-1566b (Oyster Tissue) and SRM-1577b (Bovine Liver) were obtained from the National Institute of Standards and Technology (NIST, USA). Microbial media, including Plate Count Agar (PCA), Potato Dextrose Agar (PDA), Eosin Methylene Blue Agar (EMB, Oxoid, UK), and Xylose Lysine Deoxycholate Agar (XLD, Liofilchem, Italy), were purchased from Oxoid (UK). *Pseudomonas* Selective Agar (Liofilchem, Italy) was supplied by Liofilchem (Italy). Ultrapure water (18.2 MΩ·cm) from a Milli-Q system (Millipore) was used for all dilutions and solution preparations.

### Sample Collection and Preparation

A total of 60 fresh eggs, comprising 30 poultry eggs and 30 duck eggs, were randomly collected from wholesale markets in Dhaka City, Bangladesh. The sample size was selected based on the requirements of the microbiological, physicochemical, elemental, and structural analyses and on sample sizes used in comparable egg microbiology and decontamination studies. Similar investigations have used 60 eggs for microbiological profiling and three to six eggs per treatment for decontamination and egg-quality measurements [4, 10]. At the time of purchase, eggs were visually inspected to ensure their shells were intact and free of cracks. According to vendor information, the eggs were produced within 24–48 h prior to collection, ensuring freshness. After collection, the samples were transported to the laboratory and stored at room temperature (25 ± 2 °C) before experimental treatment. The samples were divided into two main groups: poultry eggs and duck eggs. For analysis, egg components were labeled as follows: PBY (Poultry Brown Yolk), PWA (Poultry White Albumen), PWE (Poultry Whole Egg),DBY (Duck Brown Yolk), DWA (Duck White Albumen), and DWE (Duck Whole Egg).

### Sample Preparation for Elemental Analysis

Eggs from each group were manually separated into yolk, albumen, and whole egg fractions. Each fraction was microwave-dried at temperatures below 100 °C until a constant weight was achieved. Dried samples were cooled in a desiccator, finely ground using an acid-washed agate mortar and pestle, and sieved through a 2-mm nylon mesh to obtain homogeneous powders. The homogenized samples were stored in pre-cleaned polyethylene bags at − 20 °C until analysis.

### Sodium Hypochlorite Treatment

**Fig. 1:**
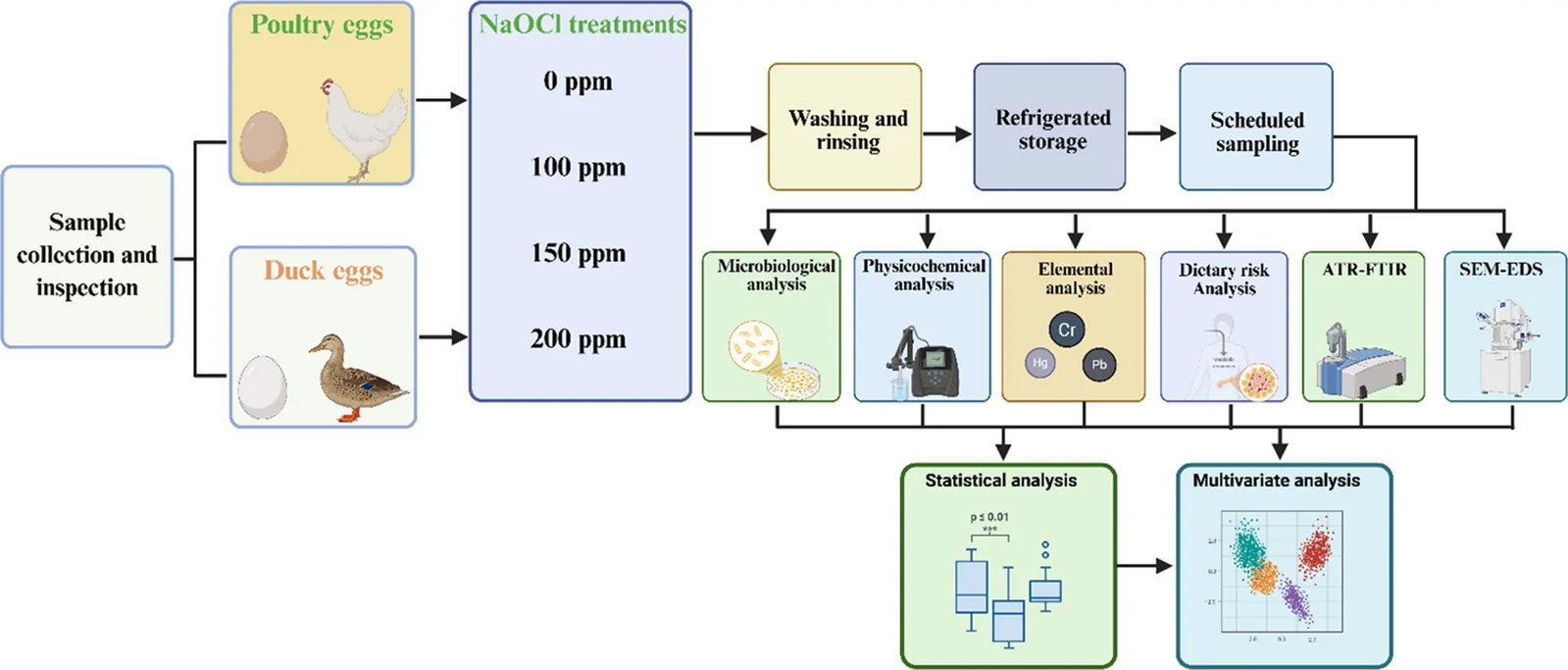
Schematic overview of the experimental design for evaluating NaOCl-treated poultry and duck eggs

For each egg type, samples were randomly assigned to four treatment groups (0, 100, 150, and 200 ppm NaOCl). Unless otherwise stated, three independent eggs per treatment were used for structural and analytical measurements, while six eggs per treatment were used for weight-loss analysis and 30 eggs per species were used for microbiological prevalence assessment. Sodium hypochlorite solutions were freshly prepared and standardized iodometrically before use. To minimize cross-contamination, eggs were washed individually in fresh sanitizer solution (500 mL per egg) for 2 min. After treatment, each egg was rinsed with fresh sterile water for 30 s, air-dried, and stored at 4 °C with 75 ± 5% relative humidity (Fig. 1). The rinsing step was performed equally across all treatments, including the control, to ensure that microbial reduction was due to NaOCl treatment rather than physical rinsing. Eggs were then used for microbial, physicochemical, elemental, and structural analyses.

### Elemental Analysis

#### Instrumental Neutron Activation Analysis (INAA)

INAA was conducted at the 3-MW TRIGA Mark II reactor (Reactor and Neutron Physics Division, AERE, Savar, Dhaka). Approximately 200 mg of each dried sample and certified reference materials (SRM-1566b Oyster Tissue and SRM-1577b Bovine Liver) were sealed in polyethylene vials [25]. Short-term irradiation (1 min at 250 kW, neutron flux: 1.77 × 10^12^ cm⁻^2^ s⁻^1^) targeted short-lived radionuclides of the elements (Al, Ca, Cl, K, Mn, Na), while long-term irradiation (7 min at 2.4 MW, neutron flux: 1.70 × 10^13^ cm⁻^2^ s⁻^1^) was used for elements such as Br, Cr, Fe, and Zn. Gamma spectra were acquired using an HPGe detector coupled to a digital spectrum analyzer, with decay and counting times optimized per element [46].

#### Atomic Absorption Spectrometry (AAS)

Atomic absorption spectrometric analysis was performed using double-deionized water and analytical-grade reagents (E. Merck, Germany). To prepare standard solutions, 1000 mg/L stock solutions were diluted. A microwave system was used to digest about 1 g of egg and reference samples (SRM-1577b, SRM-1566b) in Teflon containers with 10 ml of HNO₃ (65%) and 2 ml of H₂O₂ (30%). The digests were diluted to volume and filtered with Whatman No. 42 [52]. Flame atomic absorption spectrometry (FAAS) was used for Cu and Ni analysis, graphite furnace atomic absorption spectrometry (GFAAS) for Pb and Cd, and cold vapor atomic absorption spectrometry (CVAAS) for Hg. Instrument performance and data quality were verified using replicates, spikes, and blanks, spike recoveries ranged from 85 to 99%.

### Quality Assurance and Control

Analytical accuracy and precision were validated using SRM-1566b and SRM-1577b, analyzed by both INAA and AAS. Analytical performance was evaluated using Z-scores calculated from the measured and reference values of the certified reference materials. The Z-score was calculated according to the following Eq. (1) [37]:

1 $$\mathrm{Z}-\mathrm{s}\mathrm{c}\mathrm{o}\mathrm{r}\mathrm{e}=\frac{{X}_{lab - {X}_{ref}}}{{\sigma}_{ref}}$$

where *X*_*lab*_ is the measured value, *X*_*ref*_ the reference value, and *σ*_*ref*_ its uncertainty [36]. Results were classified as satisfactory (|Z|≤ 2), questionable (2 <|Z|< 3), or unsatisfactory (|Z|≥ 3). Accuracy and precision of the reported data by replicate analysis (*n* = 3) of SRM-1566b and SRM-1577b by INAA and AAS are presented in Table S1 (Supplementary data).

### Health Risk Assessment

#### Estimated Daily Intake (EDI)

The estimated daily intake (EDI) of Cd, Cr, Cu, Fe, Mn, Ni, Pb, and Zn was calculated following Rahman and Islam [44] using this Eq. (2):

2 $$\mathrm{E}\mathrm{D}\mathrm{I}=\frac{MC \times CF \times FIR}{BW \times 1000}$$

where *MC* is the mean concentration of metals in the samples (mg/kg), *CF* is the conversion factor (0.208), *FIR* is the daily food ingestion rate (g/person/day), and *BW* is body weight (adult = 60 kg, child = 16 kg). According to the Household Income and Expenditure Survey [21], the FIR values for the Bangladeshi population were 11.3 g (adults) and 6.5 g (children) for poultry eggs, and 8.2 g (adults) and 3.6 g (children) for duck eggs.

#### Target Hazard Quotient (THQ)

The target hazard quotient (THQ) was used to assess non-carcinogenic health risk associated with egg consumption. THQ was determined as the ratio of estimated exposure dose to the oral reference dose (RfD) Eq. (3):

3 $$\mathrm{T}\mathrm{H}\mathrm{Q}=\frac{EDI}{RfD}$$

RfD values (mg/kg/day) were obtained from [55] and [47]: As (3E-04), Cd (1E-03), Hg (5E-04), Pb (4E-03), Zn (3E-01), Cu (4E-02), Mn (1.4E-01), Ni (2E-02), Fe (7E-01), and Cr (15E-1). A THQ less than 1 indicates no significant health risk, while a THQ greater than 1 suggests a potential non-carcinogenic concern [47].

#### Target Carcinogenic Risk (TCR)

Target carcinogenic risk (TCR) values were calculated following [44] using this Eq. (4):

4 $$\mathrm{T}\mathrm{C}\mathrm{R}=\mathrm{C}\mathrm{P}\mathrm{F}\mathrm{o}\times \mathrm{E}\mathrm{D}\mathrm{I}$$

Target carcinogenic risk (TCR) was calculated using the carcinogenic potency slope factor (CPFo) and the estimated daily intake (EDI). The slope factors for Cr, Cd, and Pb were 0.41, 6.1, and 8.5 × 10⁻^3^ (mg kg⁻^1^ body weight day⁻^1^)⁻^1^, respectively [14]. The United States Environmental Protection Agency (U. S. EPA) considers the TCR below 1.0 × 10^−6^ as an "acceptable" risk level, whereas it becomes "serious" or "unacceptable" when the value exceeds 1.0 × 10^−4^ [13].

### Physicochemical Properties

#### pH

The pH of homogenized egg samples was measured using a calibrated digital pH meter (HI-2020, Hanna Instruments) at 25 ± 1 °C. The instrument was calibrated daily with certified buffer solutions [46]. Triplicate readings were taken for each sample, and the mean value was reported.

#### Weight Loss

The weight loss percentage of eggs during storage was measured at 28-day intervals using the method described by Pan et al. [42]. The eggs were weighed individually using a digital scale with a precision of 0.0001 g. Six independent egg samples were used for each treatment, and the weight loss was calculated using the following equation:

5 $$\mathrm{W}\mathrm{e}\mathrm{i}\mathrm{g}\mathrm{t} \mathrm{l}\mathrm{o}\mathrm{s}\mathrm{s} (\mathrm{\%})=\frac{Initial Weight \left(W1\right)-Final Weight \left(W2\right)}{Inital Weight \left(W1\right)}\times 100$$

### Microbial Analysis

#### Total Bacterial Count (TBC)

The total aerobic mesophilic bacterial count was determined using the standard plate count method (ISO 4833–1:2013). Each egg was aseptically rinsed in a sterile plastic bag containing 50 mL of sterile 0.1% peptone water (Merck, Germany) for 2 min with manual agitation. After microbial sampling, the eggs were aseptically cracked, and the shells were rinsed, air-dried, and weighed using an analytical balance to determine eggshell mass. The rinse was serially diluted (tenfold) in peptone water, and 1 mL aliquots of appropriate dilutions were pour-plated in duplicate onto Plate Count Agar (PCA, Oxoid). After solidification, plates were inverted and incubated at 37 ± 1 °C for 48 ± 3 h [45]. Colonies were counted manually using a Quebec colony counter, and results were expressed as log₁₀ colony-forming units per gram of eggshell (log₁₀ CFU g⁻^1^). Plates containing 30–300 colonies were used for calculations.

#### Total Fungal Count (TFC)

Fungal contamination was assessed in accordance with ISO 21527–1:2008. Eggshell rinses were similarly diluted and surface-plated (0.1 mL) onto acidified Potato Dextrose Agar (PDA, Oxoid, pH 3.5 ± 0.1 adjusted with 10% tartaric acid). Plates were incubated at 25 ± 1 °C for 5 days, after which yeasts and molds were enumerated separately based on colony morphology [48]. Results were expressed as log₁₀ CFU g⁻^1^. For quality control, blank samples (sterile peptone water) were processed simultaneously to monitor environmental contamination.

#### Bacterial Detection via Selective Culture

Selective culture techniques were used to detect *Escherichia coli*, *Salmonella* spp., and *Pseudomonas* spp. Eggshell rinses (1 mL; see Sect. "Total Bacterial Count (TBC)") were plated onto Eosin Methylene Blue agar (EMB, Oxoid) for *E. coli*, Xylose Lysine Deoxycholate agar (XLD, Oxoid) for *Salmonella*, and Cetrimide agar (Oxoid) for *Pseudomonas*. These plates were incubated at 37 °C for 24–48 h. Presumptive colonies were identified based on characteristic morphology and color (metallic green sheen on EMB for *E. coli*; red colonies with black centers on XLD for *Salmonella*; greenish colonies on Cetrimide for *Pseudomonas*) and confirmed through routine biochemical tests, including Gram staining and oxidase or catalase reactions [45]. A total of 30 poultry and 30 duck egg samples were tested. Bacterial presence was recorded as positive or negative for each target group, and prevalence was shown as the percentage of positive samples. The detection limit was 10 CFU g⁻^1^.

### Structural and Surface Characterization

#### Attenuated Total Reflectance Fourier Transform Infrared (ATR-FTIR) Spectroscopy

Eggshell membrane samples were analyzed on a Nicolet iS™10 FTIR spectrometer (Thermo Fisher) equipped with a diamond ATR accessory. Spectra were recorded from 4000 to 500 cm⁻^1^ at 4 cm⁻^1^ resolution, with 32 scans averaged per sample under a continuous N₂ purge. Between measurements, the crystal was cleaned with isopropanol and dried with N₂ [49, 63]. Data were baseline-corrected and normalized to the amide I band (~ 1650 cm⁻^1^) using OMNIC™ software. All analyses were performed in triplicate.

#### Scanning Electron Microscopy (SEM) and Energy Dispersive X-ray Spectroscopy (EDS) Analysis

Membrane samples were fixed in 2.5% glutaraldehyde, dehydrated through an ethanol series, critical-point dried, and sputter-coated with Pt (5 nm). Imaging was performed on a Hitachi Regulus 8230 FE-SEM at 5 kV, with a working distance of 8–10 mm [16]. EDS mapping and quantification were performed with an Oxford X-MaxN detector (AZtec software) at 15 kV, with a live time of 60 s.

### Statistical Analysis

Data are presented as mean ± standard deviation. Homogeneity of variance was evaluated using Levene’s test. For datasets satisfying the homogeneity assumption (*p* > 0.05), one-way analysis of variance (ANOVA) followed by Duncan’s multiple range test was used for mean comparisons. When the homogeneity assumption was violated (*p* < 0.05), Welch’s ANOVA followed by the Games–Howell post-hoc test was applied. Differences were considered statistically significant at *p* < 0.05. Statistical analyses were performed using SPSS version 25.0. Multivariate analyses (PCA, HCA) and heatmaps were generated with OriginPro 2023. All experiments were conducted in triplicate unless otherwise stated.

## Results and Discussion

### Elemental Concentrations of Poultry and Duck Eggs

The elemental analysis of poultry and duck eggs, specifically yolk, albumen, and whole egg, is presented in Table 1. Elemental accumulation in eggs mainly depends on continuous exposure through feed and water, egg origin, and species-specific differences in metal absorption and retention [23, 40]. In this study, 15 chemical elements (Al, Br, Ca, Cd, Cl, Cr, Cu, Fe, Hg, K, Mn, Na, Ni, Pb, and Zn) were measured in poultry and duck egg samples. The average concentrations of these elements in whole poultry eggs, in descending order, are: K > Cl > Na > Ca > Fe > Al > Zn > Br > Cu > Pb > Mn > Cr > Ni > Cd > Hg,and in duck eggs: K > Cl > Na > Ca > Fe > Al > Zn > Br > Cu > Pb > Mn > Ni > Cr > Cd > Hg. The concentrations (mg/kg) ranged from Al (43.1–122), Br (2.72–61.8), Ca (708–2540), Cd (< 0.050–0.097), Cl (1360–6270), Cr (< 0.04–1.29), Cu (1.90–8.88), Fe (6.84–127), Hg (< 0.020), K (2720–18700), Mn (0.790–4.63), Na (1420–3550), Ni (< 0.80–3.09), Pb (1.34–6.60), and Zn (3.97–82.3). The measured elemental concentrations were then compared to the Maximum Acceptable Concentrations (MACs) established by the Joint FAO/WHO Expert Committee on Food Additives (JECFA).

**Table 1:** The mass fractions (mg/kg) of chemical elements in egg samples

**Samples**	**Al**	**Br**	**Ca**	**Cd**	**Cl**	**Cr**	**Cu**	**Fe**
PBY	55.1 ± 1.2	2.72 ± 0.07	876 ± 50	< 0.050	4180 ± 120	< 0.04	3.22 ± 0.24	108 ± 16
PWA	61.3 ± 1.4	11.2 ± 0.23	1140 ± 60	< 0.050	1360 ± 370	0.89 ± 0.10	5.62 ± 0.10	6.84 ± 3.13
PWE	122 ± 3	14.9 ± 0.11	1640 ± 70	0.078 ± 0.010	4170 ± 120	1.29 ± 0.06	8.88 ± 0.62	127 ± 26
DBY	43.1 ± 1.3	18.9 ± 0.34	708 ± 45	0.052 ± 0.010	1150 ± 120	0.66 ± 0.04	1.90 ± 0.11	108 ± 16
DWA	51.2 ± 1.6	35.7 ± 0.63	2330 ± 90	< 0.050	4140 ± 310	< 0.04	5.21 ± 0.24	6.84 ± 3.13
DWE	101 ± 2	61.8 ± 0.31	2540 ± 50	0.097 ± 0.030	6270 ± 170	1.25 ± 0.18	7.69 ± 0.40	125 ± 17
MAC				0.1^A^		1.0^B^	10^C^	17.6^C^
**Samples**	**Hg**	**K**	**Mn**	**Na**	**Ni**	**Pb**	**Zn**	
PBY	< 0.020	2720 ± 1.2	2.17 ± 0.17	1480 ± 90	< 0.80	1.34 ± 0.10	76.3 ± 4.5	
PWA	< 0.020	12200 ± 300	1.17 ± 0.13	1680 ± 760	< 0.80	3.14 ± 0.14	3.97 ± 0.49	
PWE	< 0.020	16800 ± 400	4.03 ± 0.45	2130 ± 180	1.10 ± 0.01	4.89 ± 0.22	82.3 ± 9.9	
DBY	< 0.020	3530 ± 140	3.03 ± 0.21	2210 ± 100	< 0.80	1.88 ± 0.10	70.7 ± 4.2	
DWA	< 0.020	12900 ± 300	0.790 ± 0.100	1420 ± 640	1.38 ± 0.01	4.44 ± 0.32	5.06 ± 0.57	
DWE	< 0.020	18700 ± 700	4.63 ± 1.41	3550 ± 260	3.09 ± 0.14	6.60 ± 0.55	74.9 ± 8.5	
MAC	0.05^D^		6.5^E^			0.1^A^	100^E^	

Given values are means of different samples, and uncertainties associated with mean values are the standard deviation (1σ), except for egg samples. For egg samples, uncertainties are due to the uncertainty budget estimated for INAA

^A^JECFA [27], ^B^FAO/WHO [15], ^C^USDA [54], ^D^USDA [53], ^E^WHO [59]

*MAC* Maximum Allowable Concentration

Aluminum (Al) levels ranged from 43.1 ± 1.3 mg/kg in duck yolk (DBY) to 122 ± 3 mg/kg in poultry whole egg (PWE), indicating higher accumulation in whole eggs. Bromine (Br) and calcium (Ca) were notably elevated in duck whole eggs (61.8 ± 0.31 mg/kg Br; 2540 ± 50 Ca), consistent with efficient mineral retention during yolk–albumen integration. The Ca content aligns with values reported by Cristea et al. [9]. Bromine mainly originates from contaminated feed, water, or environmental exposure [17]. Cadmium (Cd) remained < 0.05 mg/kg in most samples except PWE (0.078 ± 0.010 mg/kg) and DWE (0.097 ± 0.030 mg/kg), all within MAC limits. Cadmium contamination commonly arises from wastewater irrigation, industrial effluents, fossil-fuel combustion, and phosphate fertilizers, which transfer Cd to feed and drinking water [46]. Chronic Cd exposure can impair kidney function, reduce egg production, and cause gastrointestinal toxicity [47].

Iron (Fe) concentrations were notably higher in poultry eggs, reaching 127 ± 26 mg/kg in poultry whole eggs (PWE). Comparable iron concentrations have been reported in poultry eggs from Bangladesh [4]. Such enrichment may be attributed to iron-rich poultry feed and elevated groundwater iron levels in the Dhaka region [7, 52]. Although iron is an essential micronutrient involved in oxygen transport, enzymatic activity, and immune function, excessive dietary intake may be associated with adverse health effects [47]. Chlorine (Cl) and chromium (Cr) were highest in DWE (6270 ± 170 Cl,1.25 ± 0.18 mg/kg Cr). Cr concentrations in PWE and DWE exceeded the MAC. Potential sources include tannery waste incorporated into feed and environmental contamination [44]. High Cr exposure can cause oxidative stress, respiratory irritation, and carcinogenic effects. Copper (Cu) ranged from 1.90 ± 0.11 to 8.88 ± 0.62 mg/kg, all within safe limits. Cu is essential for enzyme activity but becomes toxic at excessive levels [52]. Mercury (Hg) was below detection (< 0.020 mg/kg) in all samples, indicating negligible contamination.

Potassium (K) was substantially higher in duck eggs (up to 18700 ± 700 mg/kg) and lowest in poultry yolk, reflecting species-specific differences in electrolyte content. K, Mn (up to 4.63 ± 1.41 mg/kg), and Na (up to 3550 ± 260) were also higher in duck whole eggs, confirming their greater mineral density. These elements play essential roles in nerve function, fluid balance, bone development, and antioxidant defense [23, 46]. Lead (Pb) levels were elevated in all samples, especially in PWE (4.89 ± 0.22 mg/kg) and DWE (6.60 ± 0.55 mg/kg), exceeding MAC limits. Pb contamination typically arises from polluted feed, water, fuel combustion, and industrial emissions [4]. Chronic Pb exposure harms neurological, renal, and cardiovascular systems in humans and induces oxidative stress in poultry [46]. Similar Pb levels have been reported in eggs from Bangladesh [51]. Although Pb and Cr concentrations exceeded the maximum allowable concentrations in some samples, the calculated dietary exposure indices (EDI, THQ, and TCR) remained within acceptable limits due to relatively low egg consumption rates. Zinc (Zn) levels were 82.3 ± 9.9 mg/kg in PWE and 74.9 ± 8.5 mg/kg in DWE, both within MAC limits. Zn supports enzymatic and immune functions but can be harmful at excessive levels [51].

### Elemental Correlations Matrix

**Fig. 2:**
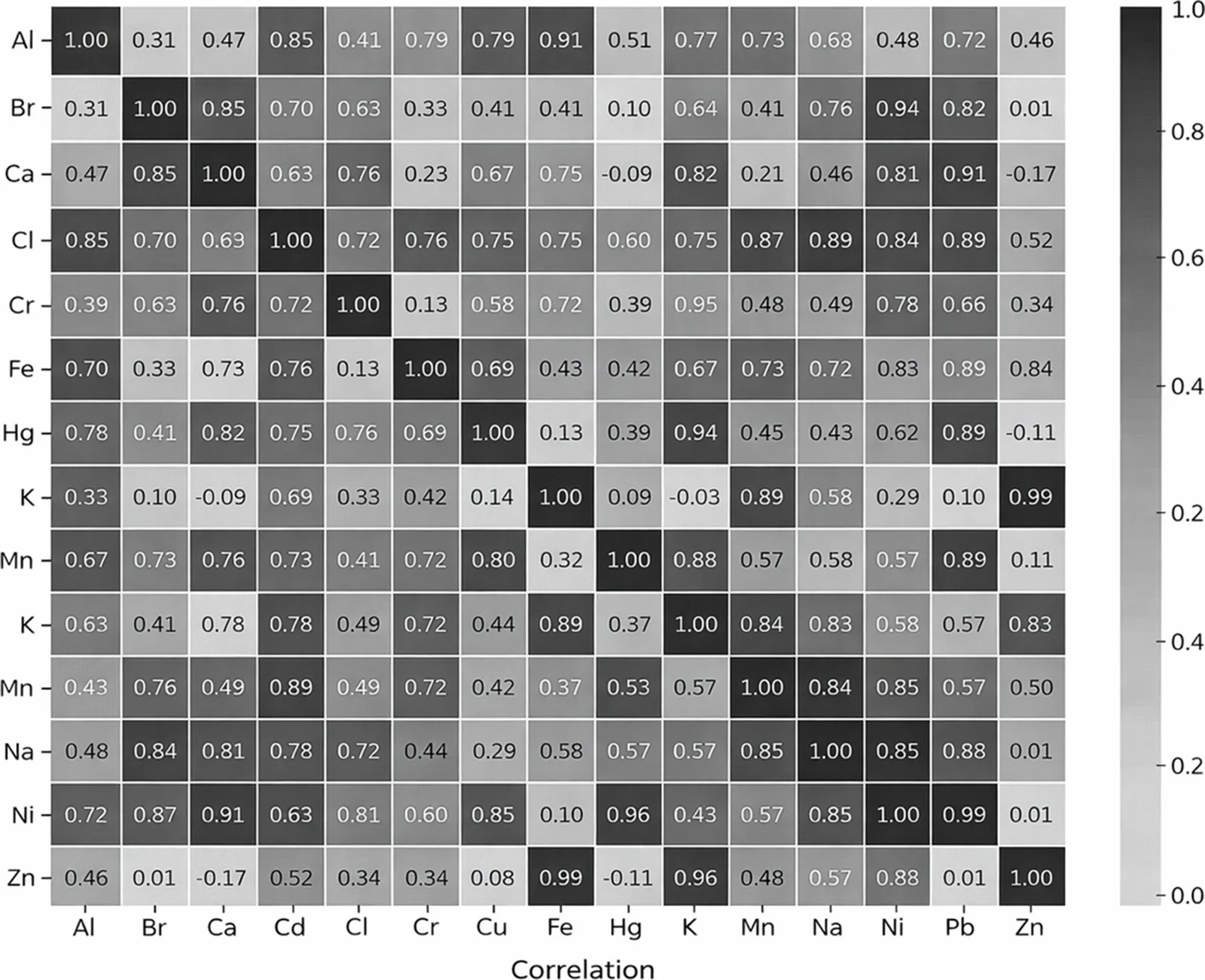
Correlation matrix heatmap illustrating the relationships among elemental concentrations in the analyzed samples

The Pearson correlation matrix revealed distinct clustering among elemental concentrations in poultry and duck eggs (Fig. 2). Elements such as Fe, Zn, Cu, K, Ni, Mn, and Pb showed moderate to strong positive correlations, indicating similar accumulation patterns most likely driven by shared dietary inputs and co-exposure during egg formation rather than by identical metabolic pathways [4]. Fe displayed an almost perfect correlation with Zn (*r* ≈ 0.99), consistent with their coordinated deposition in association with metalloproteins and enzyme systems, whereas its association with Cu was weak (*r* ≈ 0.14), suggesting more distinct regulatory control for Cu. Strong positive correlations between K and Cu (*r* ≈ 0.94) and between K and Ni (*r* ≈ 0.67) indicate concurrent incorporation during mineral deposition in developing eggs [23]. Moderate to high correlations, such as Ca–Cd (*r* ≈ 0.63) and Cd–Mn (*r* ≈ 0.87), support the view that Cd competes with essential divalent cations for transport and binding sites, a pattern also reported for interactions between Cd and Fe/Mn in biological tissues. Cl, Br, and Cr, on the other hand, had poor or inconsistent associations (*r* < 0.50) with the majority of elements, suggesting that fluctuating environmental inputs (such as local water, soil, or feed pollution) have a greater influence on their levels than strictly regulated metabolic pathways [9]. According to earlier reports on egg and poultry products, Pb, which has no physiological function, mainly reflects environmental contamination from feed, water, and surrounding matrices,its correlations with essential elements probably result from common exposure pathways rather than from homeostatic regulation [4, 30, 29].

### Multivariate Statistical Analysis

**Fig. 3:**
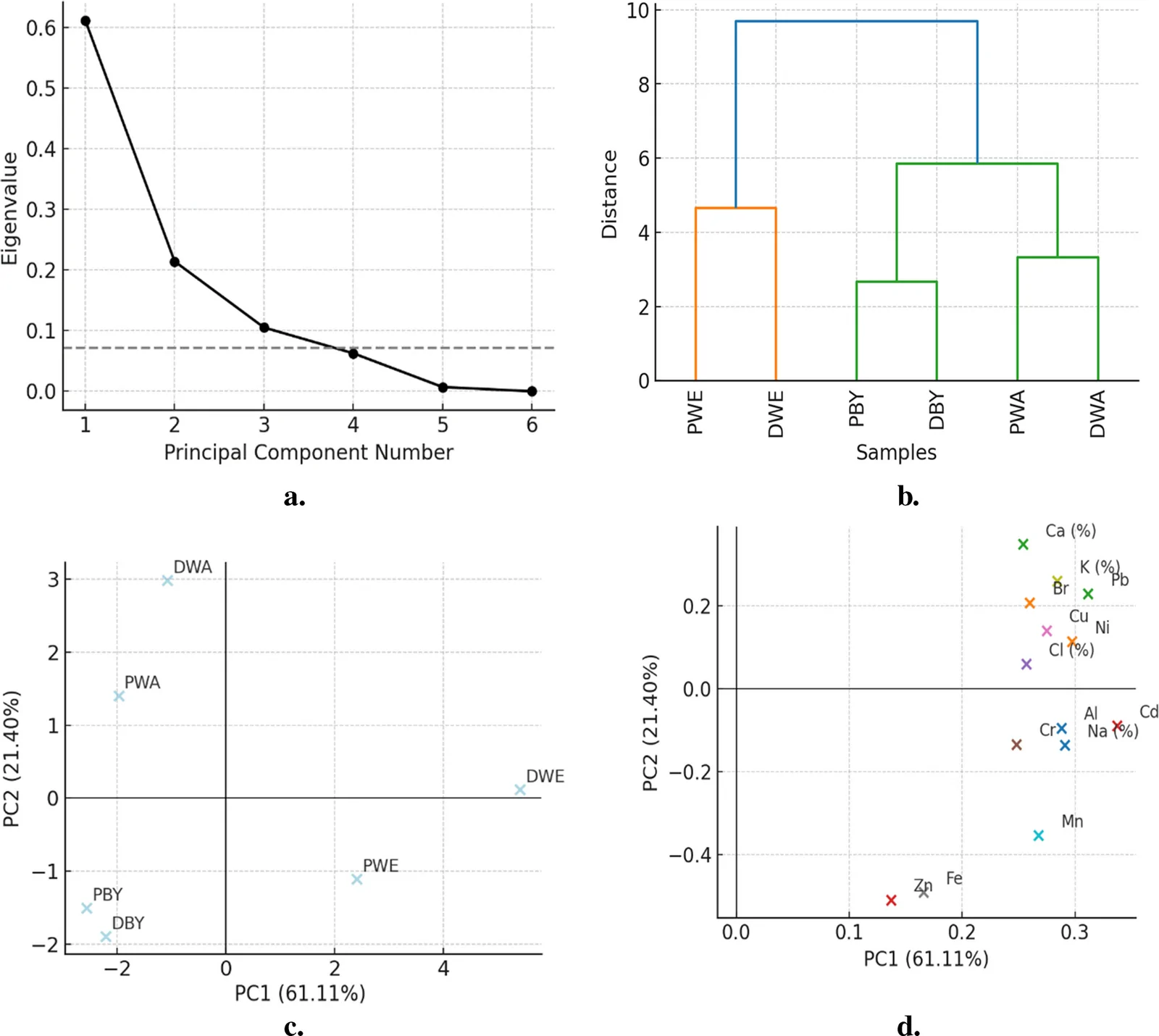
Multivariate statistical analysis of egg samples: **a** Scree plot of eigenvalues, **b** HCA dendrogram of sample clustering, **c** PCA score plot of samples, and **d** PCA loading plot of elements

Multivariate statistical analyses, including principal component analysis (PCA) and hierarchical cluster analysis (HCA), were employed to examine relationships and grouping patterns in poultry and duck egg matrices (Fig. 3a–d). The scree plot (Fig. 3a) displayed a sharp decline in eigenvalues after PC2, indicating that PC1 and PC2 adequately summarized the data. Together, these components explained 82.5% of the total variance (PC1 = 61.11%; PC2 = 21.40%). PC1 primarily differentiated samples based on higher levels of essential and trace elements, while PC2 captured variations related to minor and toxic metals such as Cd and Cr. The chemometric procedures adhered to established guidelines [62], and similar trends have been observed in other studies [4]. The HCA dendrogram (Fig. 3b) grouped the six samples into two main clusters, consistent with previously reported elemental distribution patterns in poultry eggs [51]. Cluster I included whole egg samples from both species (DWE and PWE), which exhibited relatively higher levels of Fe, Zn, and Cu. Cluster II consisted of yolk and albumen samples (PBY, DBY, PWA, and DWA), showing lower yet more consistent elemental profiles. The close relationship between PWA and DWA indicates a strong similarity between the albumen matrices, while DWE emerged as the most distinct branch, reflecting its unique elemental composition.

The PCA score plot (Fig. 3c) confirmed these groupings. DWE and PWE were clearly separated along the positive PC1 axis due to higher elemental accumulation, while PWA and DWA were positioned along the negative PC2 axis, indicating lower mineral-binding capacity. Yolk samples (PBY and DBY) clustered near the origin, suggesting intermediate and more balanced elemental levels. The PCA loading plot (Fig. 3d) revealed two main elemental groupings: essential nutrients (Fe, Zn, Cu, Mn, Ca, and K) loaded positively on PC1, whereas toxic elements (Cd, Cr, and Pb) were mainly associated with PC2, emphasizing differences in their biological roles and deposition pathways [29]. This separation highlights how essential and non-essential elements differently contribute to variability in egg matrices.

### Human Health Risk Assessment

#### Estimated Daily Intake (EDI) of Heavy Metals

The estimated daily intake (EDI) of eight trace metals (Fe, Zn, Cu, Mn, Cd, Cr, Ni, and Pb) from various egg types was calculated to evaluate dietary exposure risks (Table 2). EDI values, shown as mg/kg body weight per day, were higher in children than in adults when adjusted for body weight, due to their lower body weight and comparatively higher intake per unit of mass. For instance, EDI values for Fe and Zn in children were approximately 1.36 × 10⁻^2^ and 1.56 × 10⁻^2^ mg/kg body weight per day, respectively, exceeding those seen in adults.

**Table 2:** Comparison of the estimated daily intake (EDI) of heavy metals from eggs with the corresponding maximum tolerable daily intake (MTDI)

Samples		Estimated daily intake (mg kg⁻^1^ bw day⁻^1^)							
		Cd	Cr	Cu	Fe	Mn	Ni	Pb	Zn
PBY	Adult	ND	ND	6.31E-5	2.10E-3	4.25E-5	ND	2.62E-5	1.49E-3
	Child	ND	ND	1.36E-4	4.60E-3	9.17E-5	ND	6.00E-5	3.22E-3
PWA	Adult	ND	1.74E-5	1.10E-4	1.30E-4	2.29E-5	ND	6.15E-5	7.78E-5
	Child	ND	3.76E-5	2.37E-4	3.00E-4	4.94E-5	ND	1.30E-4	1.70E-4
PWE	Adult	3.06E-6	5.05E-5	3.50E-4	5.00E-3	1.60E-4	4.31E-5	1.90E-4	3.22E-3
	Child	6.60E-6	1.09E-4	7.50E-4	1.07E-4	3.40E-4	9.00E-5	4.13E-4	6.95E-3
DBY	Adult	7.39E-7	9.38E-6	2.70E-5	1.50E-3	4.31E-5	ND	2.67E-5	1.00E-3
	Child	1.22E-6	1.50E-5	4.40E-5	2.50E-3	7.00E-5	ND	4.40E-5	1.65E-3
DWA	Adult	ND	ND	7.41E-5	9.72E-5	1.12E-5	1.96E-5	6.31E-5	7.19E-5
	Child	ND	ND	1.22E-4	2.00E-4	2.00E-5	3.00E-5	1.04E-4	1.20E-4
DWE	Adult	2.76E-6	3.55E-5	2.20E-4	3.60E-3	1.30E-4	8.78E-5	1.90E-4	2.13E-3
	Child	4.54E-6	5.90E-5	3.60E-4	5.90E-3	2.20E-4	1.40E-4	3.09E-4	3.51E-3
Total	Adult	6.55E-6	1.12E-4	8.44E-4	1.24E-2	4.09E-4	1.50E-4	5.57E-4	7.98E-3
Total	Child	1.23E-5	2.20E-4	1.64E-3	1.36E-2	7.91E-4	2.60E-4	1.06E-3	1.56E-2
MTDI		0.66^c^	2.8^a^	1.67^c^	-	-	4.3^b^	3.0^c^	-

(^a^[50], ^b^[58] and ^c^[26] *ND* Not detected (below detection limit)

However, all EDI values for both adults and children stayed below their respective oral reference doses (RfD), indicating no significant health risk. Similarly, the EDI values for toxic elements such as Cd, Cr, Ni, and Pb were also significantly below their safety thresholds. These findings show that consuming the studied eggs results in minimal dietary exposure to trace elements and remains within the safety limits set by the Joint FAO/WHO Expert Committee on Food Additives (JECFA).

#### Non-Carcinogenic Risk

The Target Hazard Quotient (THQ) approach was used to assess non-carcinogenic health risks from elemental exposure through egg consumption, while the Hazard Index (HI) represented the cumulative risk from multiple metals (Table 3). All individual THQ values were less than 1, indicating no significant non-carcinogenic health risk from the analyzed elements. The metals followed the THQ trend: Cu > Fe > Zn > Mn > Pb > Cr > Ni > Cd. Among the samples, poultry whole eggs (PWE) and duck whole eggs (DWE) showed the highest total THQ values, with children demonstrating greater vulnerability (maximum TTHQ = 1.74 × 10⁻^1^ for PWE). This increased susceptibility in children is linked to their lower body weight and higher intake-to-body-mass ratios. The HI values for adults (2.21 × 10⁻^1^) and children (4.21 × 10⁻^1^) remained well below the safety threshold (HI < 1), confirming that current egg consumption levels do not pose a significant chronic health risk.

**Table 3:** Target hazard quotient (THQ) values of the elements for consuming egg samples

Samples		Target hazard quotient (THQ)								
		Cd	Cr	Cu	Fe	Mn	Ni	Pb	Zn	TTHQ
PBY	Adult	ND	ND	1.58E-3	3.02E-3	3.04E-4	ND	6.56E-3	4.98E-3	1.64E-02
	Child	ND	ND	3.40E-3	6.51E-3	6.55E-4	ND	1.41E-2	1.07E-2	3.54E-02
PWA	Adult	ND	1.16E-5	2.75E-3	1.91E-4	1.64E-4	ND	1.54E-2	2.59E-4	1.88E-02
	Child	ND	2.50E-5	5.93E-3	4.12E-4	3.53E-4	ND	3.31E-2	5.59E-4	4.04E-02
PWE	Adult	3.06E-3	3.36E-5	8.69E-3	7.10E-3	1.12E-3	2.15E-3	4.78E-2	1.07E-2	8.07E-02
	Child	6.59E-3	7.26E-5	1.87E-2	1.53E-2	2.43E-3	4.64E-3	1.03E-1	2.31E-2	1.74E-01
DBY	Adult	7.39E-4	6.25E-6	6.75E-4	2.19E-3	3.08E-4	ND	6.68E-3	3.34E-3	1.39E-02
	Child	1.21E-3	1.03E-5	1.11E-3	3.61E-3	5.06E-4	ND	1.09E-2	5.51E-3	2.29E-02
DWA	Adult	ND	ND	1.85E-3	1.39E-4	8.02E-5	9.81E-4	1.58E-2	2.40E-4	1.91E-02
	Child	ND	ND	3.04E-3	2.28E-4	1.32E-4	1.61E-3	2.59E-2	3.94E-4	3.13E-02
DWE	Adult	2.75E-3	2.36E-5	5.46E-3	5.07E-3	9.40E-4	4.39E-3	4.69E-2	7.09E-3	7.26E-02
	Child	4.54E-3	3.90E-5	8.99E-3	8.35E-3	1.54E-4	7.23E-3	7.72E-2	1.16E-2	1.18E-01
Total	Adult	1.68E-04	1.50E-08	4.48E-04	4.60E-04	6.58E-03	1.66E-04	3.66E-05	6.75E-04	2.21E-01 (HI)
Total	Child	3.13E-04	2.93E-08	8.73E-04	8.78E-04	9.80E-03	2.98E-04	6.86E-05	1.29E-05	4.21E-01 (HI)

*ND* Not detected (Below detection limit)

#### Target Carcinogenic Risk (TCR) Assessment

**Fig. 4:**
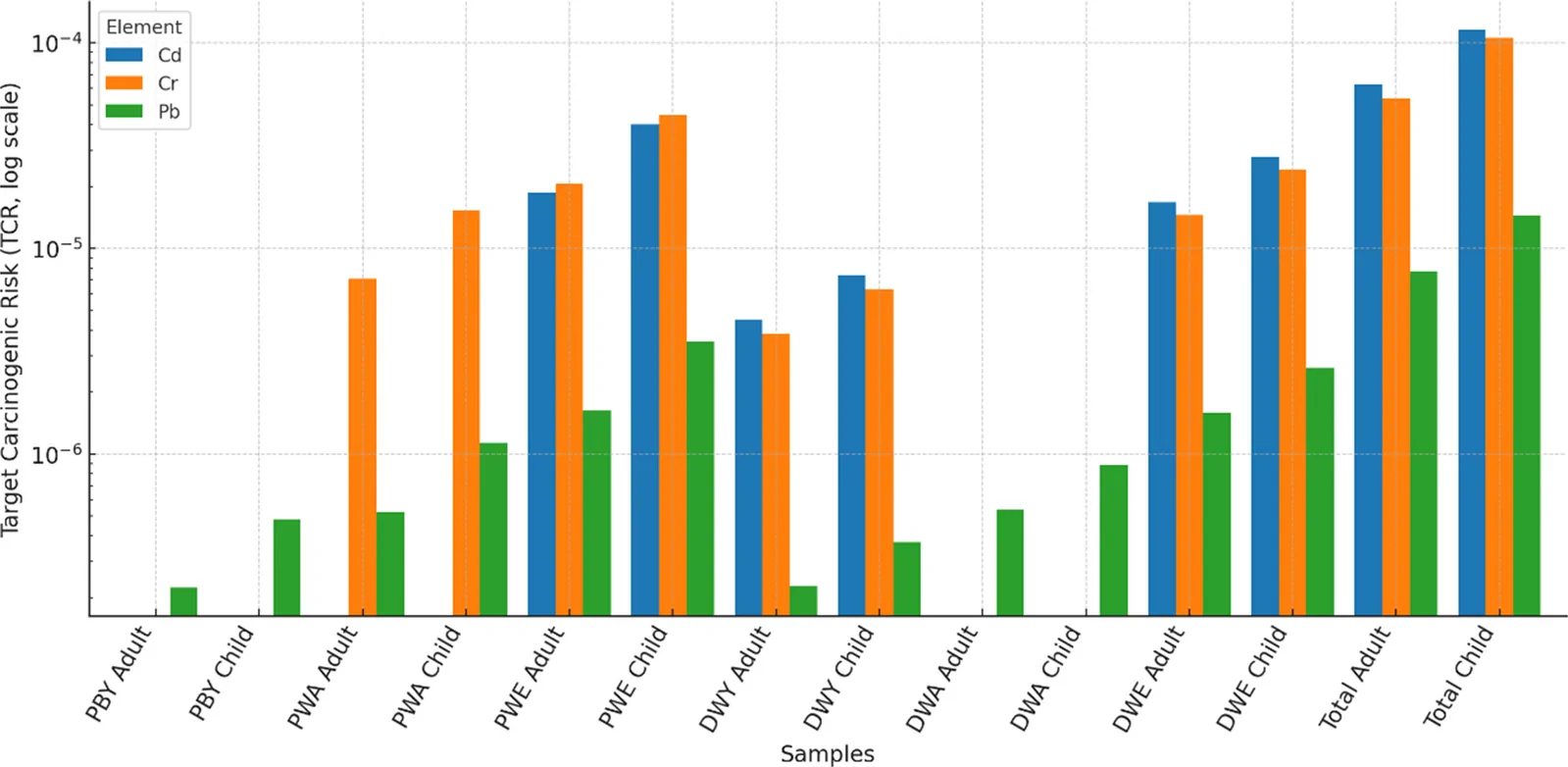
Target carcinogenic risk (TCR) of Cd, Cr, and Pb in egg samples for adults and children

The target carcinogenic risk (TCR) values for adults and children were calculated based on the concentrations of Cr, Pb, and Cd in the analyzed egg samples and are shown in Table 4. All measured elements had TCR values below the acceptable risk limit of 1.0 × 10⁻^4^, indicating a negligible carcinogenic risk from egg consumption under current dietary exposure conditions [47]. The total TCR values ranged from 5.38 × 10⁻^5^ to 1.06 × 10⁻^4^ for Cr, 7.72 × 10⁻⁶ to 1.44 × 10⁻^5^ for Pb, and 6.28 × 10⁻^5^ to 1.16 × 10⁻^4^ for Cd across all egg varieties for both adults and children (Fig. 4). Although these values stay within the tolerable risk range (10⁻⁶–10⁻^4^) recommended by the U.S. EPA, the relatively higher TCR values related to Cr and Cd in poultry and duck eggs underline the importance of continuous monitoring of feed sources, especially those potentially contaminated with industrial or tannery by-products [46, 52]. Overall, the results indicate that current egg consumption carries minimal carcinogenic risk,however, ongoing monitoring is advised to prevent potential increases in long-term exposure.

**Table 4:** Target carcinogenic risk (TCR) of the elements due to consumption of poultry and eggs sample

Samples	Cd		Cr		Pb	
	Adult	Child	Adult	Child	Adult	Child
PBY	ND	ND	ND	ND	2.23E-7	4.81E-7
PWA	ND	ND	7.15E-6	1.54E-5	5.22E-7	1.13E-6
PWE	1.86E-5	4.02E-5	2.07E-5	4.47E-5	1.62E-6	3.51E-6
DBY	4.51E-6	7.42E-6	3.85E-6	6.33E-6	2.27E-7	3.74E-7
DWA	ND	ND	ND	ND	5.36E-7	8.83E-7
DWE	1.68E-5	2.77E-5	1.46E-5	2.40E-5	1.59E-6	2.62E-6
Total	6.28E-5	1.16E-4	5.38E-5	1.06E-4	7.72E-6	1.44E-5

### Physicochemical Properties

At 4 °C, the pH of both poultry whole eggs (PWE) and duck whole eggs (DWE) increased gradually over 28 days, reflecting CO₂ loss and albumen alkalization (Table 5). The pH of PWE rose from 7.3 ± 0.02 to 7.5 ± 0.06, while DWE increased from 7.5 ± 0.02 to 7.8 ± 0.04. This mild alkalization results from the natural breakdown of carbonic acid and the subsequent diffusion of CO₂ from the albumen, which decreases hydrogen-ion concentration [3, 60]. Duck eggs consistently had higher pH values than chicken eggs, likely because of their thicker shells and lower gas permeability [34]. Egg weight changes were evaluated during storage at different concentrations of NaOCl. At 0 ppm, poultry eggs weighed 66.16 ± 4.33 g, whereas at 200 ppm, the weight decreased to 61.82 ± 4.08 g after 28 days. Similarly, duck eggs decreased from 74.22 ± 3.41 g to 71.88 ± 4.88 g (Table 6). This reduction may result from the oxidative action of NaOCl, which can partially degrade the eggshell’s cuticle and calcium carbonate matrix, causing gradual moisture and mineral loss during storage [41, 57]. Moderate NaOCl concentrations (100–150 ppm) maintained acceptable shell strength while reducing microbial contamination without causing excessive structural damage.

**Table 5:** pH values of egg samples during storage at 4 ^0^C for 28 days

Samples	0 Days	7 Days	14 Days	21 Days	28 Days
PWE	7.30 ± 0.10^c^	7.33 ± 0.06^bc^	7.43 ± 0.06^ab^	7.43 ± 0.12^ab^	7.53 ± 0.06^a^
DWE	7.47 ± 0.06^c^	7.57 ± 0.06^bc^	7.57 ± 0.03^bc^	7.60 ± 0.05^b^	7.73 ± 0.12^a^

Values are expressed as mean ± SD (*n* = 3). Different superscript letters within each row indicate significant differences according to Duncan’s Multiple Range Test (DMRT, *p* < 0.05)

**Table 6:** Effect of sodium hypochlorite (NaOCl) concentrations on egg weight (g) during storage at 4 °C

Days	0 ppm	100 ppm	150 ppm	200 ppm
0—Poultry	66.16 ± 4.33^a^	59.12 ± 2.89^c^	62.95 ± 4.09^b^	61.82 ± 4.08^b^
7	65.59 ± 4.07^a^	58.32 ± 2.99^c^	62.50 ± 3.84^b^	61.70 ± 4.29^b^
14	65.45 ± 3.55^a^	58.13 ± 2.55^c^	62.32 ± 3.75^b^	61.55 ± 3.88^b^
21	64.97 ± 3.72^a^	56.84 ± 2.88^c^	61.86 ± 4.80^b^	61.09 ± 4.15^b^
28	63.23 ± 4.07^a^	56.42 ± 3.05^c^	61.06 ± 2.99^b^	59.88 ± 2.89^b^
0—Duck	74.22 ± 3.41^b^	75.09 ± 4.22^b^	73.39 ± 4.25^a^	71.88 ± 4.88^a^
7	74.20 ± 3.40^a^	75.00 ± 2.80^a^	73.39 ± 3.88a^b^	71.78 ± 4.19^b^
14	74.00 ± 3.50^b^	74.88 ± 2.25^b^	73.25 ± 3.55^a^	71.75 ± 3.80^a^
21	73.92 ± 3.77^b^	74.75 ± 2.80^a^	72.75 ± 4.09^a^	70.25 ± 4.01^a^
28	73.50 ± 4.20^b^	73.65 ± 3.35^ab^	71.36 ± 2.99^a^	69.10 ± 2.99^a^

Values are expressed as mean ± SD (*n* = 3). Different superscript letters within the same row indicate significant differences among NaOCl concentrations according to Duncan’s Multiple Range Test (DMRT, *p* < 0.05)

### Microbial Analysis

The total bacterial and fungal counts in poultry egg samples were 7.0 × 10⁷ CFU g⁻^1^ and 1.4 × 10^4^ CFU g⁻^1^, respectively, while duck eggs showed slightly higher initial loads of 8.4 × 10⁷ CFU g⁻^1^ and 1.8 × 10^4^ CFU g⁻^1^ (Table 7). According to recommendations from the European Union, FAO, and the United States, acceptable total bacterial counts (TBC) in eggs or egg-based products typically range from 10^5^ to 10⁶ CFU g⁻^1^ [8]. Untreated poultry eggs showed detection rates of presumptive *E. coli* (26.66%), presumptive *Salmonella* spp. (16.66%), and presumptive *Pseudomonas* spp. (10%), while duck eggs exhibited slightly higher prevalence levels of 33.33%, 23.33%, and 10%, respectively. After treatment with 100 ppm sodium hypochlorite, presumptive *E. coli* and presumptive *Salmonella* spp. were reduced to 16.66% and 6.66% in poultry eggs, and to 16.66% and 10% in duck eggs, respectively, while presumptive *Pseudomonas* spp. were not detected.

**Table 7:** Effect of Sodium Hypochlorite (NaOCl) concentrations on microbial contamination reduction of eggshell

Conc. (ppm)	TBC (CFU/g)	Log₁₀ Reduction	TFC (CFU/g)	Log₁₀ Reduction	*E. coli spp (n* = *30)*	*Salmonella spp (n* = *30)*	*Pseudomonas spp (n* = *30)*
0—Poultry	(7.0 ± 0.5) × 10⁷	ND	(1.4 ± 0.2) × 10^4^	ND	33.33%	26.66%	16.66%
100	(2.8 ± 0.2) × 10^4^	3.40	(7.0 ± 0.3) × 10^2^	1.30	16.66%	6.66%	ND
150	< 10 CFU/g	≥ 6.85	< 10 CFU/g	≥ 3.15	ND	ND	ND
200	< 10 CFU/g	≥ 6.85	< 10 CFU/g	≥ 3.15	ND	ND	ND
0—Duck	(8.4 ± 0.6) × 10⁷	ND	(1.8 ± 0.3) × 10^4^	ND	46.66%	33.33%	13.33%
100	(3.2 ± 0.3) × 10^4^	3.42	(9.0 ± 0.2) × 10^2^	1.30	23.33%	10.0%	ND
150	< 10 CFU/g	≥ 6.92	< 10 CFU/g	≥ 3.25	ND	ND	ND
200	< 10 CFU/g	≥ 6.92	< 10 CFU/g	≥ 3.25	ND	ND	ND

*ND* Not detected (< 10 CFU/g). For samples below the detection limit, log₁₀ reductions are reported as minimum values (≥)

Additionally, treatment with 100 ppm NaOCl significantly reduced microbial loads in both egg types, achieving approximately 3.4 log₁₀ reductions in total bacterial counts and 1.3 log₁₀ reductions in fungal counts. Similar findings have been reported by Damena et al. [10], who observed aerobic mesophilic bacterial loads ranging from 1.22 log₁₀ CFU mL⁻^1^ to 9.70 log₁₀ CFU mL⁻^1^ on eggshell rinsates and from 1.52 log₁₀ CFU mL⁻^1^ to 9.36 log₁₀ CFU mL⁻^1^ in internal egg contents. Increasing NaOCl concentrations (100–200 ppm) further decreased bacterial and fungal counts, reflecting the strong oxidative action of NaOCl, which forms hypochlorous acid capable of disrupting microbial cell membranes, proteins, and nucleic acids [24, 42]. At 150 and 200 ppm, no cultivable bacteria or fungi were detected above the detection limit (10 CFU g⁻^1^) on either poultry or duck eggshells, indicating effective surface decontamination. Essential oils are also used to decontaminate the egg surface. Recent studies have reported significant microbial reductions following essential-oil treatment of eggshells, although efficacy depends on oil composition, concentration, exposure method, and storage conditions [12, 56]. Potential effects on odor, flavor, and consumer acceptance should also be considered before commercial application.

### Morphological and Elemental Characterization

#### FTIR Spectroscopic Analysis

**Fig. 5:**
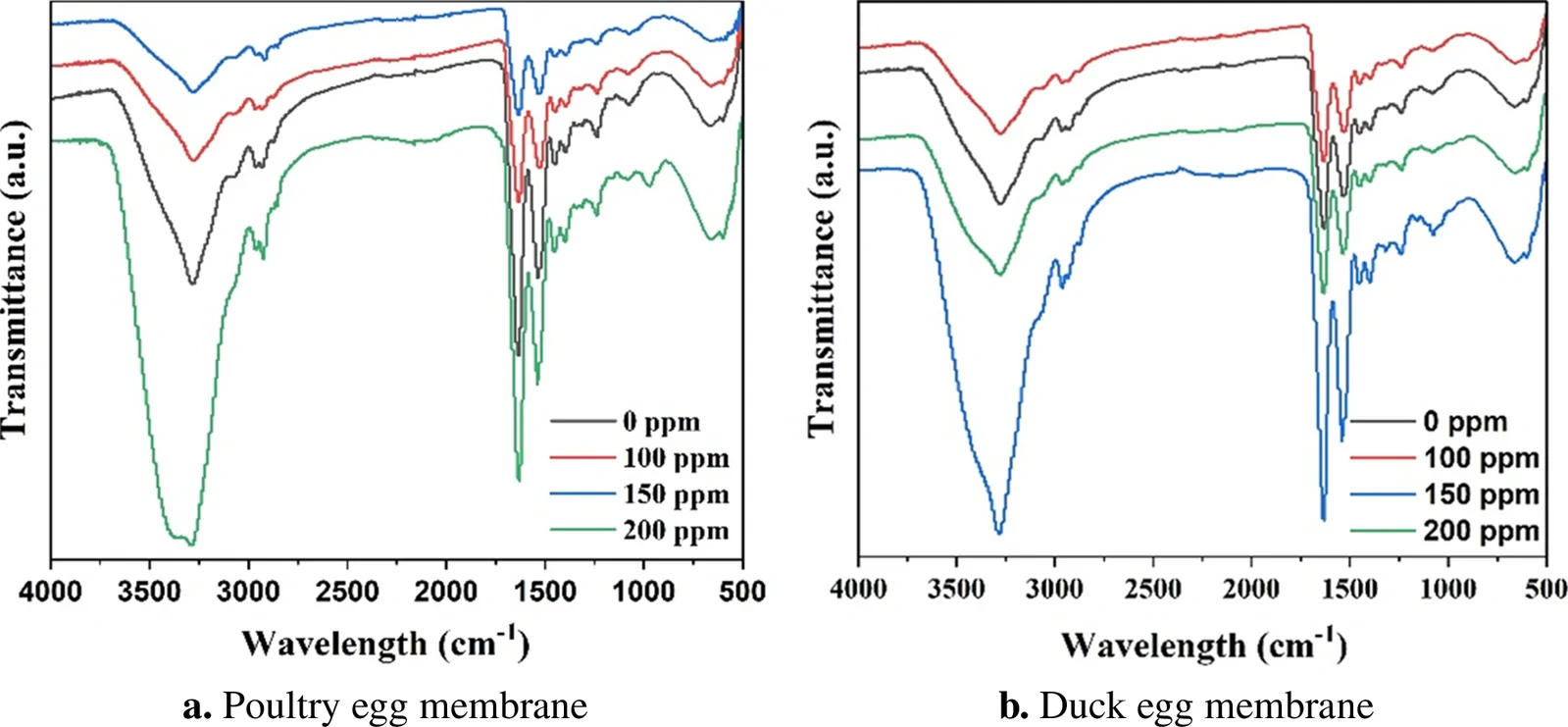
Fourier transform infrared (FTIR) spectra of inner eggshell membranes from poultry (**a**) and duck (**b**) eggs treated with sodium hypochlorite (NaOCl) at 0, 100, 150, and 200 ppm, showing concentration-dependent structural changes

FTIR spectra of poultry and duck eggshell membranes showed significant chemical and structural changes after NaOCl treatment (Fig. 5). The broad O–H/N–H stretching band at 3300–3400 cm⁻^1^ increased with higher NaOCl concentrations, indicating protein oxidation and structural modifications related to exposure of hydroxyl and amine groups [63]. In untreated membranes, clear amide I (~ 1650 cm⁻^1^) and amide II (~ 1550 cm⁻^1^) bands reflected intact peptide structures,these bands gradually weakened and shifted following NaOCl treatment, suggesting modifications of peptide bonds and partial changes in secondary protein structure [22]. The decrease in bands within the 1000–1200 cm⁻^1^ range indicates alterations in glycoprotein and carbohydrate-related functional groups. Duck eggshell membranes exhibited stronger amide bands at higher NaOCl levels, suggesting possible greater resistance to oxidative modification compared to poultry membranes [33].

#### Scanning Electron Microscopy Analysis

**Fig. 6:**
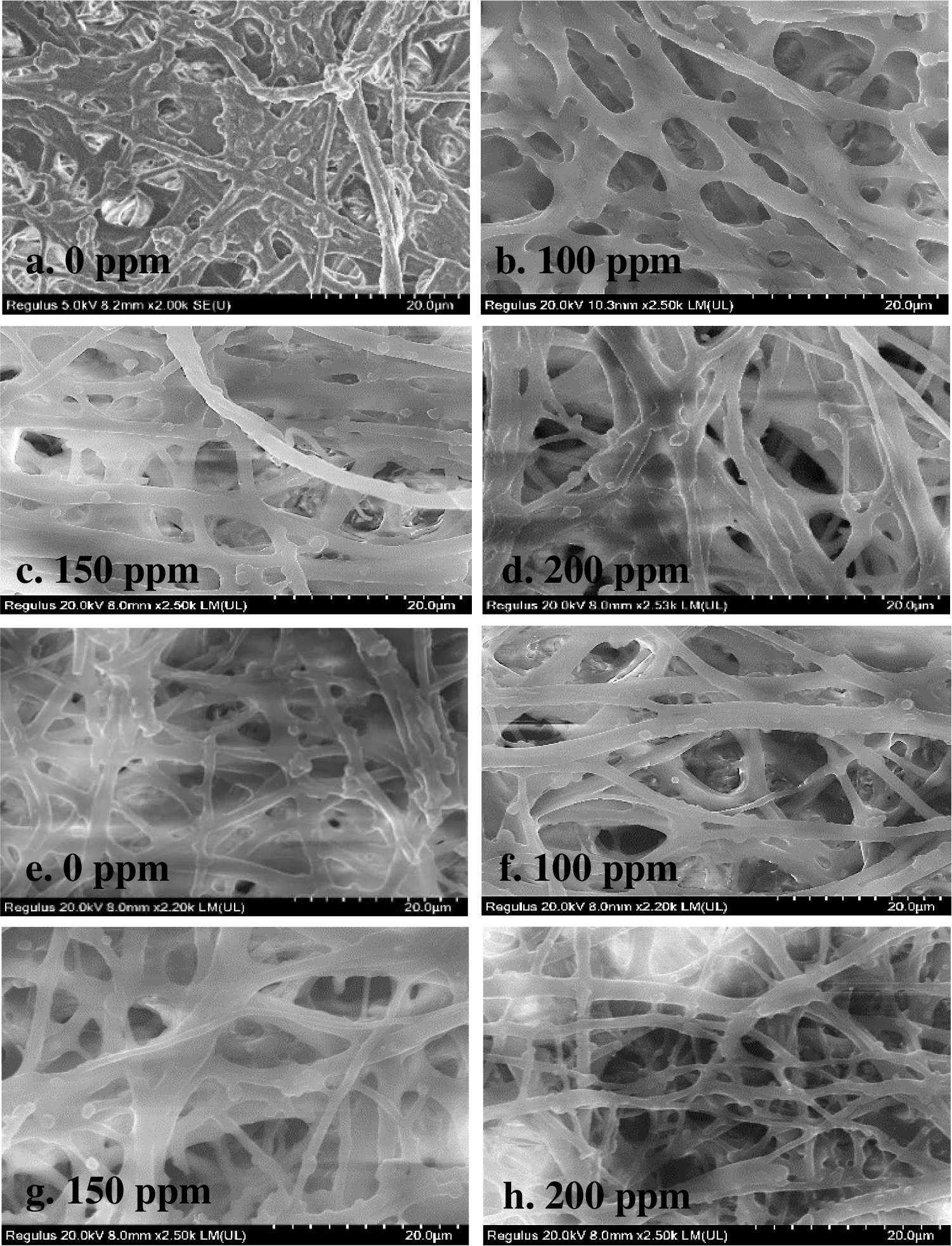
Scanning electron micrographs of inner eggshell membranes from poultry (**a**–**d**) and duck (**e**–**h**) eggs treated with different concentrations of sodium hypochlorite (NaOCl; 0, 100, 150, and 200 ppm)

SEM images of the inner eggshell membranes of ducks and poultry at various NaOCl concentrations (0–200 ppm) and their changes are shown in Fig. 6. Smooth, continuous fibrils indicated an intact protein matrix, and both untreated membranes displayed a dense, organized collagen fiber mesh typical of the interwoven insoluble protein network in eggshell membranes and cuticle structures. A similar result was also reported by Han et al. [19]. At 100 ppm, slight fiber loosening and pore expansion appeared, indicating early oxidative effects on protein cross-links. Similar to the oxidative breakdown and denaturation of structural proteins observed under more aggressive physicochemical treatments, collagen bundles became noticeably thinner and partially fragmented at a concentration of 150 ppm, indicating significant disruption of the fibrous structure under oxidative stress [22]. Widened pores and collapsed fibers indicated severe deterioration at 200 ppm. Due to differences in collagen density and cross-linking, poultry membranes showed relatively greater structural disruption under the tested conditions to hypochlorite-induced oxidation than duck membranes. Higher concentrations (> 150 ppm) caused severe membrane damage, while lower concentrations (≤ 100 ppm) resulted in minimal structural changes, as seen in the SEM images.

#### Energy Dispersive X-Ray Spectroscopy (EDS)

EDS analysis revealed that eggshell membranes consist of the major elements carbon, nitrogen, and oxygen, confirming their protein- and collagen-rich composition, as presented in Table 8. Elemental mapping showed a uniform distribution of these organic elements, while Pt signals, originating from sputter coating during SEM preparation, are shown in supporting Figure S1. NaOCl treatment resulted in slight compositional changes. Carbon levels remained relatively stable (56.2–58.8 wt%), whereas nitrogen slightly increased (18.9–19.9 wt%) and oxygen decreased, accompanied by an increase in sulfur (3.7–4.6 wt%), consistent with possible oxidative modification of sulfur-containing amino acids, consistent with previous observations in oxidized protein matrices [35]. Chlorine and sodium contents also rose (Cl: 0.7–1.1 wt%,Na: 0.5–0.8 wt%), reflecting adsorption of chlorine-based sanitizer residues on the membrane surface [24, 42]. These elemental shifts align with the FTIR findings for NaOCl-treated eggshell membranes and support oxidative modification of the membrane structure during sanitization [31].

**Table 8:** EDS analysis (wt%) of poultry egg membranes treated with different concentrations of Sodium Hypochlorite (NaOCl)

Conc. (ppm)	C	N	O	S	Pt	Cl	K	Na	Al	Ca	Mg
0	56.2 ± 2.8	18.9 ± 0.8	17.9 ± 0.3	3.7 ± 0.3	1.1 ± 0.01	0.7 ± 0.02	0.7 ± 0.02	0.5 ± 0.04	0.2 ± 0.01	0.1 ± 0.0	ND
150	58.8 ± 2.6	19.6 ± 0.5	12.9 ± 0.6	4.6 ± 0.2	1.5 ± 0.03	0.9 ± 0.04	0.8 ± 0.03	0.7 ± 0.02	0.3 ± 0.02	ND	0.1 ± 0.0
200	56.3 ± 2.7	19.9 ± 0.7	14.9 ± 0.4	4.2 ± 0.1	1.4 ± 0.05	1.1 ± 0.01	0.9 ± 0.01	0.8 ± 0.02	0.5 ± 0.01	ND	ND

*ND* Not detected (below the practical EDS detection limit of approximately 0.1 wt% for Na, Mg, Al, Ca, K, and Cl, and 0.5 wt% for C, N, and O)

#### Limitations of this Study

This study has several limitations. First, a formal a priori power analysis was not conducted, and three independent eggs were used for some structural and analytical endpoints; therefore, these findings should be interpreted as laboratory-scale evidence. Second, eggs were obtained from wholesale markets in a single geographic area, which may limit generalization to other breeds, production systems, and regions. Third, the controlled washing and storage conditions may not fully replicate commercial processing and distribution. Fourth, only selected NaOCl concentrations and one contact time were evaluated, and free chlorine, chlorate, perchlorate, and other possible chlorination by-products were not directly quantified. Fifth, presumptive bacterial isolates were identified using selective culture and routine biochemical tests without molecular confirmation. Finally, sensory quality and consumer acceptance were not evaluated. Future studies should include an a priori sample-size calculation, larger multi-location sampling, commercial-scale validation, molecular pathogen confirmation, direct analysis of chlorine-related residues and by-products, and sensory assessment.

## Conclusion

This study showed that washing with sodium hypochlorite (NaOCl) effectively enhances the microbial safety of poultry and duck eggs, while its effect on eggshell membrane integrity depends heavily on concentration. NaOCl treatment reduced total bacterial and fungal contamination, and concentrations of 150–200 ppm lowered cultivable microorganisms below detectable levels. However, FTIR, SEM, and EDS analyses indicated that higher NaOCl concentrations gradually changed membrane chemistry, disrupted collagen fibril organization, and increased surface chlorination. Conversely, moderate treatment levels caused less structural damage while still significantly reducing microbes. Elemental analysis also revealed that although Pb and Cr levels exceeded maximum allowable concentrations in some samples, the estimated daily intake (EDI), target hazard quotient (THQ), hazard index (HI), and target carcinogenic risk (TCR) stayed within acceptable safety limits for both adults and children under the typical consumption scenario. These results suggest that NaOCl washing is an effective egg sanitation method, but the concentration must be carefully controlled to balance microbial decontamination with maintenance of membrane structure and egg quality.

## Supplementary Information

Below is the link to the electronic supplementary material.

Supplementary file1 (DOCX 1720 KB)

## Data Availability

No datasets were generated or analysed during the current study.

## References

[1] Bangladesh Bureau of Statistics (BBS) (2023) Household income and expenditure survey 2022. Bangladesh Bureau of Statistics, Dhaka, Bangladesh

[2] Bermudez-Aguirre D, Niemira BA (2023) A review on egg pasteurization and disinfection: traditional and novel processing technologies. Compr Rev Food Sci Food Saf 22(2):756–784 10.1111/1541-4337.13088 36537903

[3] Biesek J, Wlaźlak S, Adamski M (2023) Changes in physicochemical parameters of duck eggs and extra-embryonic structures during incubation. Animal 17(12):101024 10.1016/j.animal.2023.101024 37981451

[4] Biswas M, Annana RP, Kundu GK, Hossain MP, Rahman AH, Alam M (2025) Estimation of avy metals in egg varieties: a potential risk assessment for human health. Food Control 171:111107

[5] Cardoso MJ, Nicolau AI, Borda D, Nielsen L, Maia RL, Møretrø T, Teixeira P (2021) *Salmonella* in eggs: from shopping to consumption-a review providing an evidence-based analysis of risk factors. Compr Rev Food Sci Food Saf 20(3):2716–2741 10.1111/1541-4337.12753 33960652

[6] Chen X, Wang J, Han J, Yang X, Zhu L, Luo X, Liang R (2025) Effects of ultrasound-assisted sodium hypochlorite on the decontamination and storage quality of chilled chicken leg-quarters. Food Control 168:110895

[7] Choudhury N, Siddiqua TJ, Ahmed ST, Haque MA, Ali M, Dil Farzana F, Ahmed T (2022) Iron content of drinking water is associated with anaemia status among children in high groundwater iron areas in Bangladesh. Trop Med Int Health 27(2):149–157 10.1111/tmi.13710 34905267

[8] Chousalkar KK, Khan S, McWhorter AR (2021) Microbial quality, safety and storage of eggs. Curr Opin Food Sci 38:91–95

[9] Cristea G, Covaciu FD, Feher I, Puscas R, Voica C, Dehelean A (2024) Multivariate modelling based on isotopic, elemental, and fatty acid profiles to distinguish the backyard and barn eggs. Foods 13(20):3240 10.3390/foods13203240 PMC11507348 39456302

[10] Damena A, Mikru A, Adane M, Dobo B (2022) Microbial profile and safety of chicken eggs from a poultry farm and small-scale vendors in Hawassa, Southern Ethiopia. J Food Qual 2022(1):7483253. 10.1155/2022/7483253

[11] Eissa FI, Shehata AM (2024) Eggs and egg products contamination: analysis of the EU RASFF notifications from 2000 to 2022. Food Control 158:110249

[12] El-Soufi A, Al Khatib A, Khazaal S, El Darra N, Raafat K (2025) Evaluation of essential oils as natural antibacterial agents for eggshell sanitization and quality preservation. Processes 13(1):224

[13] EPA U (2000) Supplementary guidance for conducting health risk assessment of chemical mixtures. Washington DC: USA.

[14] Epa, U. S. (2011) Exposure factors handbook. Off. Res. Dev., Washington, DC 20460, p pp 2-6

[15] FAO/WHO (2002) Codex Alimentarius—general standards for contaminants and toxins in food. Schedule 1 Maximum and Guideline levels for contaminants and toxins in food. In: Joint FAO/WHO Food Standards Programme, Codex Committee. Rotterdam, Reference CX/FAC 02/16.

[16] Fathi MB, RahmatAbadi ZT (2024) Eggshell microstructure, shell quality indices, mineralogy, and UV–Vis absorbance of domestic eggs of Iran. J Photochem Photobiol 21:100235

[17] Fernandes AR, Lake IR, Dowding A, Rose M, Jones NR, Smith F, Panton S (2023) The transfer of environmental contaminants (brominated and chlorinated dioxins and biphenyls, PBDEs, HBCDDs, PCNs and PFAS) from recycled materials used for bedding to the eggs and tissues of chickens. Sci Total Environ 892:164441 10.1016/j.scitotenv.2023.164441 37245822

[18] Guyonnet V (2025) Advances in shell egg safety regulations and protocols. Advances in improving the safety and quality of eggs. Burleigh Dodds Science Publishing, Cambridge, UK, pp 79–114

[19] Han C, Chen Y, Shi L, Chen H, Li L, Ning Z, Wang D (2023) Advances in eggshell membrane separation and solubilization technologies. Front Vet Sci 10:1116126 10.3389/fvets.2023.1116126 PMC10060898 37008347

[20] Henchion M, Moloney AP, Hyland J, Zimmermann J, McCarthy S (2021) Trends for meat, milk and egg consumption for the next decades and the role played by livestock systems in the global production of proteins. Animal 15:100287 10.1016/j.animal.2021.100287 34312092

[21] HIES (Household Income and Expenditure Survey) (2011) Preliminary report on household income and expenditure survey2010. Bangladesh Bureau of Statistics, Statistics Division, Ministry of Planning, Dhaka, Bangladesh

[22] Homavand A, Cree DE, Wilson LD (2024) Investigation of eggshell membrane removal with two different treatment methods: thermal and chemical. Adv Polym Technol 2024(1):6533629

[23] Hossain A, Ahmed MW, Rabin MH, Kaium A, Razzaque MA, Zamil SS (2024) Heavy metal quantification in chicken meat and egg: an emerging food safety concern. J Food Compos Anal 126:105876

[24] Hsu SC, Chen HL, Chou CF, Liu WC, Wu CT (2023) Characterization of microbial contamination of retail washed and unwashed shell eggs in Taiwan. Food Control 149:109718

[25] Islam MA, Das B, Quraishi SB, Khan R, Naher K, Hossain SM, Hossen MB (2020) Heavy metal contamination and ecological risk assessment in water and sediments of the Halda river, Bangladesh: a natural fish breeding ground. Mar Pollut Bull 160:111649 10.1016/j.marpolbul.2020.111649 33181930

[26] JECFA (2003) Summary and conclusions of the 61st meeting of the Joint FAO/WHO Expert Committee on Food Additives (JECFA), JECFA/61/SC. Italy, Rome

[27] JECFA (2005) Codex General Standard for Contaminants and Toxins in Food and Feeds. In: 64th Meeting of the Joint FAO/WHO Expert Committee on Food Additives (JECFA), JECFA/64/CAC/ RCP 49–2001.

[28] Jones DR, Garcia JS, Gast RK, Ward GE (2021) Equivalency of peroxyacetic acid to chlorine as a shell egg sanitizing rinse. Poult Sci 100(6):101069 10.1016/j.psj.2021.101069 PMC8065222 33848928

[29] Khan S, Shi X, Cai R, Zhao S, Li X, Khan IM, Guo J (2024) Assessing the performance, egg quality, serum analysis, heavy metals and essential trace metals accumulation in laying hen eggs and tissues fed black soldier fly (*Hermetia illucens*) larvae meal. Poult Sci 103(12):104315 10.1016/j.psj.2024.104315 PMC11462471 39316985

[30] Korish MA, Attia YA (2020) Evaluation of heavy metal content in feed, litter, meat, meat products, liver, and table eggs of chickens. Animals 10(4):727 10.3390/ani10040727 PMC7222721 32331361

[31] Kulshreshtha G, Rodriguez-Navarro A, Sanchez-Rodriguez E, Diep T, Hincke MT (2018) Cuticle and pore plug properties in the table egg. Poult Sci 97(4):1382–1390 10.3382/ps/pex409 29340658

[32] Kulshreshtha G, Ward C, Calvert ND, Benavides-Reyes C, Rodriguez-Navarro AB, Diep T, Hincke MT (2024) Effect of egg washing and hen age on cuticle quality and bacterial adherence in table eggs. Microorganisms 12(10):2027 10.3390/microorganisms12102027 PMC11509795 39458336

[33] Liu Y, Cai Z, Ma M, Sheng L, Huang X (2020) Effect of eggshell membrane as porogen on the physicochemical structure and protease immobilization of chitosan-based macroparticles. Carbohydr Polym 242:116387 10.1016/j.carbpol.2020.116387 32564851

[34] Luo W, Xue H, Xiong C, Li J, Tu Y, Zhao Y (2020) Effects of temperature on quality of preserved eggs during storage. Poult Sci 99(6):3144–3157 10.1016/j.psj.2020.01.020 PMC7597647 32475451

[35] Mahato PL, Weatherby T, Ewell K, Jha R, Mishra B (2024) Scanning electron microscope-based evaluation of eggshell quality. Poult Sci 103(3):103428 10.1016/j.psj.2024.103428 PMC10847681 38301498

[36] Messaoudi M, Begaa S (2019) Dietary intake and content of some micronutrients and toxic elements in two Algerian spices (*Coriandrum sativum L.* and *Cuminum cyminum L*.). Biol Trace Elem Res 188(2):508–513 10.1007/s12011-018-1417-8 29911262

[37] Mkangara M (2023) Prevention and control of human *Salmonella enterica* infections: an implication in food safety. Int J Food Sci 2023(1):8899596 10.1155/2023/8899596 PMC10506869 37727836

[38] Morales-Juárez A, Cowan-Pyle AE, Bailey RL, Eicher-Miller HA (2024) Eating egg-rich diets and modeling the addition of one daily egg reduced risk of nutrient inadequacy among United States adolescents with and without food insecurity. J Nutr 154(11):3475–3484 10.1016/j.tjnut.2024.09.019 PMC11827025 39307281

[39] Movasaghi M, Heydari MM, Schwean-Lardner K, Kirychuk S, Thompson B, Zhang L (2025) Investigating cold plasma jet effectiveness for eggshell surface decontamination. Food Control 168:110928

[40] Nimako C, Khidkhan K, Kulprasertsri S, Poapolathep S, Khunlert P, Yohannes YB, Poapolathep A (2025) Pollution and partitioning of neonicotinoid insecticides in free-grazing ducks and their eggs: implications for human health. Environ Pollut 373:126099 10.1016/j.envpol.2025.126099 40120842

[41] Oliveira GDS, McManus C, Salgado CB, Dos Santos VM (2022) Effects of sanitizers on microbiological control of hatching eggshells and poultry health during embryogenesis and early stages after hatching in the last decade. Animals 12(20):2826 10.3390/ani12202826 PMC9597748 36290211

[42] Pan D, Li R, Li Y, Gao X, Fan X, Du Q, Zhou C (2022) Effects of manual washing with three alkaline sterilizing agent solutions on egg quality during storage. Food Chem 396:133733 10.1016/j.foodchem.2022.133733 35872500

[43] Rafiq K, Sani AA, Hossain MT, Hossain MT, Hadiuzzaman M, Bhuiyan MAS (2024) Assessment of the presence of multidrug-resistant *Escherichia coli*, *Salmonella* and *Staphylococcus* in chicken meat, eggs and faeces in Mymensingh division of Bangladesh. Heliyon 10(17):e36690 10.1016/j.heliyon.2024.e36690 PMC11401041 39281621

[44] Rahman M, Islam MA (2019) Concentrations and health risk assessment of trace elements in cereals, fruits, and vegetables of Bangladesh. Biol Trace Elem Res 191(1):243–253 10.1007/s12011-018-1596-3 30612302

[45] Rahman M, Islam MA, Das KC, Salimullah M, Mollah MZI, Khan RA (2021) Effect of gamma radiation on microbial load, physico-chemical and sensory characteristics of common spices for storage. J Food Sci Technol 58(9):3579–3588 10.1007/s13197-021-05087-4 PMC8292502 34366475

[46] Rahman M, Islam MA, Khan RA (2018) Characterization of chemical elements in common spices of Bangladesh for dietary intake and possible health risk assessment by INAA and AAS techniques. J Radioanal Nucl Chem 318(2):1347–1357

[47] Rahman M, Islam MA, Zaved MM (2020) Assessment of essential and potentially toxic elements and possible health risks in *Hylocereus undatus* and *Punica granatum*. Biol Trace Elem Res 198(2):707–713 10.1007/s12011-020-02072-4 32048147

[48] Rahman M, Moniruzzazan M, Das KC, Islam MA, Kamal MM, Rahman MS, Khan RA (2023) Effect of gamma radiation on fungal load decontamination of marketed spices. Arch Agric Environ Sci 8(4):468–475

[49] Rahman M, Sobhan A, Kotturi H, Sadique S, Sadak O, Khandaker M (2026) Development of antimicrobial food packaging films based on electrospun polycaprolactone nanofibers encapsulating thyme oil nanoemulsions. J Agric Food Res 26:102704

[50] RDA (1989) Recommended dietary allowance, 10th edn. National Academic Press, Washington

[51] Samad A, Roy D, Hasan MM, Ahmed KS, Sarker S, Hossain MM, Shajahan M (2023) Intake of toxic metals through dietary eggs consumption and its potential health risk assessment on the peoples of the capital city Dhaka, Bangladesh. Arab J Chem 16(10):105104

[52] Ullah AA, Afrin S, Hosen MM, Musarrat M, Ferdoushy T, Nahar Q, Quraishi SB (2022) Concentration, source identification, and potential human health risk assessment of heavy metals in chicken meat and egg in Bangladesh. Environ Sci Pollut Res Int 29(15):22031–22042 10.1007/s11356-021-17342-4 34775560

[53] USDA (2014) United States Department of Agriculture, China: China Releases the Standard for Maximum Levels of Contaminants in Foods.

[54] USDA national nutrient database for standard reference, release 24. Nutrient Data Laboratory. Retrieved from https://www.ars.usda.gov/. (Accessed 17 August 2023)

[55] USEPA (2015) Risk based screening table. Composite table: summary tab 0615. Accessible at. http://www2.epa.gov/risk/riskbasedscreeningtablegenerictables (accessed on 25 September, 2018)

[56] Vale IRR, Oliveira GDS, de Jesus LM, de Castro MB, McManus C, Dos Santos VM (2024) Sustainable bacterial control of hatching eggshells using essential oils. Antibiotics (Basel) 13(11):1025 10.3390/antibiotics13111025 PMC11591154 39596720

[57] Wales A, Taylor E, Davies R (2022) Review of food grade disinfectants that are permitted for use in egg packing centres. Worlds Poult Sci J 78(1):231–260

[58] WHO (1996) Trace elements in human nutrition and health. World Health Organization, Geneva, Switzer-land, pp 242–244

[59] WHO (1998) Environmental health criteria IPCS. International Program of Chemical Society

[60] Wlaźlak S, Brzycka Z, Ragus W, Banaszak M, Grabowicz M (2024) Quality characteristics, lysozyme activity, and albumen viscosity of fresh hatching duck eggs after a week’s storage at various temperatures. Sci Rep 14(1):5616 10.1038/s41598-024-56351-4 PMC10920898 38454129

[61] Yu HC, Chen IC, Tan FJ (2025) Evaluation of washing with sodium hypochlorite, ultraviolet irradiation, and storage temperature on shell egg quality during storage. Foods 14(13):2156 10.3390/foods14132156 PMC12248953 40646910

[62] Zaldarriaga Heredia J, Wagner M, Jofré FC, Savio M, Azcarate SM, Camiña JM (2023) An overview on multi-elemental profile integrated with chemometrics for food quality assessment: toward new challenges. Crit Rev Food Sci Nutr 63(26):8173–8193 10.1080/10408398.2022.2055527 35319312

[63] Zhai S, Zhang W, Li T, Zhang W, Lv L, Pan B (2018) Sodium hypochlorite assisted membrane cleaning: alterations in the characteristics of organic foulants and membrane permeability. Chemosphere 211:139–148 10.1016/j.chemosphere.2018.07.144 30071425

