# Supplementary material for: Effects of Sodium Hypochlorite Washing on Eggshell Membrane Integrity, Microbial Decontamination, and Trace Element-Related Dietary Risk in Poultry and Duck Eggs
Source: Biol Trace Elem Res. 2026 Aug 10;204(11):8485–501. doi: 10.1007/s12011-026-05274-4 (PMC13714127; doi:10.1007/s12011-026-05274-4)
Supplement: Supplementary file 1 [file 12011_2026_5274_MOESM1_ESM.docx]

**Table S1** Comparison of measured values with certified values (mg/kg) in the standard reference materials of SRM-1566b (Oyster tissue) and SRM-1577b (Bovine liver)

|  |  | NIST-SRM-1566b |  |  |  | NIST-SRM-1577b |  |  |  |  |
| --- | --- | --- | --- | --- | --- | --- | --- | --- | --- | --- |
| Elements | Technique | This study (n=3)^a^ | Certificate value | Ratio^b^ | Z-score | This study (n=3)^a^ | Certificate value | Ratio^b^ | Z-score | Detection limit |
| Al | INAA | 195±3 | 197.2±6.0 | 0.99 | -0.366 | - | (3)^c^ | - | - | 0.92 |
| Br | INAA | 57±1 | (55)^c^ | 1.04 | - | 8.78±0.19 | (9.7)^c^ | 0.91 | - | 2.17 |
| Ca | INAA | 808±45 | 838±20 | 0.96 | -1.5 | 107±24 | 116±4 | 0.92 | -2.25 | 6.70 |
| Cd | AAS | 2.44±0.06 | 2.48±0.08 | 0.98 | -0.5 | 0.503±0.101 | (0.5)^c^ | 1.01 | - | 0.05 |
| Cl | INAA | 5153±143 | 5140±100 | 1.00 | 0.13 | 2652±76 | 2780±60 | 0.95 | -2.13 | 1.50 |
| Cr | INAA | 0.46±0.09 | (0.51)^c^ | 0.90 | - | 0.24±0.061 | (0.307)^c^ | 0.78 | - | 0.04 |
| Cu | AAS | 70.0±2.2 | 71.6±1.6 | 0.98 | -1.0 | 159.3±3.6 | 160±8 | 1.00 | -0.087 | 0.60 |
| Fe | INAA | 217±28 | 205.8±6.8 | 1.05 | 1.65 | 165±22 | 184±15 | 0.90 | -1.26 | 1.08 |
| Hg | AAS | 0.035±0.001 | 0.0371±0.0013 | 0.94 | -1.61 | 0.002±0.001 | (0.003)^c^ | 0.67 | - | 0.02 |
| K | INAA | 6367±213 | 6520±90 | 0.98 | -1.7 | 9902±264 | 9940±20 | 1.00 | -1.9 | 1.22 |
| Mn | INAA | 18.12±0.58 | 18.5±0.2 | 0.98 | -1.9 | 9.54±0.38 | 10.5±1.7 | 0.91 | -0.56 | 1.04 |
| Na | INAA | 3178±143 | 3297±53 | 0.96 | -2.24 | 2308±105 | 2420±60 | 0.95 | -1.86 | 0.92 |
| Ni | AAS | 1.00±0.01 | 1.04±0.09 | 0.96 | -0.44 | 0.26±0.051 | (0.27)^c^ | 0.96 | - | 0.80 |
| Pb | AAS | 0.303±0.002 | 0.308±0.009 | 0.98 | -0.55 | 0.14±0.10 | (0.129)^c^ | 1.09 | - | 0.60 |
| Zn | INAA | 1508±83 | 1424±46 | 1.06 | 1.82 | 111±6 | 127±16 | 0.87 | -1.0 | 1.10 |

^a^This work values are mean values (n= 3) and uncertainties are due to standard deviation (1σ); Values in parentheses are non-certified

^b^Ratio = determined value/certified value ^c^Compiled value from GeoRem [Rahman et al., 20218]

|  |  |  |
| --- | --- | --- |
|  |  |  |
|  |  |  |

**Figure S1.** Elemental mapping of poultry egg membrane (0 ppm NaOCl) showing uniform distribution of C, O, and N, with minor Na, K, and Cl traces; Pt peaks originated from sputter coating.
